# Supplementary material for: A study of trust mining algorithms for beacon nodes in large-scale network environments
Source: PeerJ Comput Sci. 2025 Apr 22;11:e2755. doi: 10.7717/peerj-cs.2755 (PMC12190256; doi:10.7717/peerj-cs.2755)
Supplement: Supplemental Information 3 [file peerj-cs-11-2755-s003.docx]

| **Column Name** | **Description** |
| --- | --- |
| X-Axis (Rows) | This axis (rows) contains the number of nodes in increasing order. |
| Y-Axis (Columns) | This axis (columns) contains the number of cycles for experimentation. |
